# Gene Pathways That Delay *Caenorhabditis elegans* Reproductive Senescence

**DOI:** 10.1371/journal.pgen.1004752

**Published:** 2014-12-04

**Authors:** Meng C. Wang, Holly D. Oakley, Christopher E. Carr, Jessica N. Sowa, Gary Ruvkun

**Affiliations:** 1 Huffington Center on Aging, Baylor College of Medicine, Houston, Texas, United States of America; 2 Department of Molecular and Human Genetics, Baylor College of Medicine, Houston, Texas, United States of America; 3 Department of Molecular Biology, Massachusetts General Hospital, Boston, Massachusetts, United States of America; 4 Department of Genetics, Harvard Medical School, Boston, Massachusetts, United States of America; 5 Department of Earth, Atmospheric and Planetary Sciences, Massachusetts Institute of Technology, Cambridge, Massachusetts, United States of America; Stanford University School of Medicine, United States of America

## Abstract

Reproductive senescence is a hallmark of aging. The molecular mechanisms regulating reproductive senescence and its association with the aging of somatic cells remain poorly understood. From a full genome RNA interference (RNAi) screen, we identified 32 *Caenorhabditis elegans* gene inactivations that delay reproductive senescence and extend reproductive lifespan. We found that many of these gene inactivations interact with insulin/IGF-1 and/or TGF-β endocrine signaling pathways to regulate reproductive senescence, except *nhx*-2 and *sgk*-1 that modulate sodium reabsorption. Of these 32 gene inactivations, we also found that 19 increase reproductive lifespan through their effects on oocyte activities, 8 of them coordinate oocyte and sperm functions to extend reproductive lifespan, and 5 of them can induce sperm humoral response to promote reproductive longevity. Furthermore, we examined the effects of these reproductive aging regulators on somatic aging. We found that 5 of these gene inactivations prolong organismal lifespan, and 20 of them increase healthy life expectancy of an organism without altering total life span. These studies provide a systemic view on the genetic regulation of reproductive senescence and its intersection with organism longevity. The majority of these newly identified genes are conserved, and may provide new insights into age-associated reproductive senescence during human aging.

## Author Summary

Female reproductive senescence is one hallmark of human aging, and as the germline ages, there is increased incidence of chromosome non-dysjunction and DNA damage. Delayed childbearing is a general feature in modern society, resulting in high risk of infertility, miscarriage and birth defects. Thus, understanding the molecular mechanisms regulating reproductive senescence and its association with somatic senescence is increasingly relevant to human health, and will shed light on the prolongation of reproductive longevity and the improvement of post-reproductive health. Here we conducted a genomic screen in *Caenorhabditis elegans*, searching for genetic regulators of reproductive senescence. We identified 32 gene inactivations that extend reproductive lifespan. Functional characterization of these genes has revealed their interactions with insulin/IGF-1 and TGF-β signaling pathways, their effects in different genders on the regulation of reproductive longevity, and their implications in the control of healthy life expectancy. Many of these genes are conserved between worms and humans. Our studies thus provide new insights into the molecular control of reproductive aging and the mechanistic link between reproductive senescence and organism longevity.

## Introduction

Age-associated reproductive decline is one of the first aging phenotypes to manifest in humans. As women age, they experience both a decline in fertility and an increased risk of miscarriage and birth defects [1], [2], and completely cease reproduction (reproductive senesce) after menopause. The mean age of natural menopause is 50–51 years in the western world, with a variation from 40–61 years [3]. Studies of menopausal onset demonstrated an association between mothers and daughters and also between sister pairs, suggesting the involvement of genetic factors in regulating reproductive senescence [4], [5], [6]. The genetic contributions have been estimated to range from 30 to 85% [4], [5], [6]. However, the exact genes and signaling pathways that regulate the onset and progression of reproductive senescence remain largely unknown.

*C. elegans* has been proven an effective model for studying reproduction and aging processes using various genetic approaches, genome-scale screens and live microscopy imaging. Although evolutionarily distant, *C. elegans* and humans share many conserved molecular pathways. *C. elegans* also undergoes reproductive senescence and ceases reproducing progeny after one third of their lifespan [7]. Like the increase in chromosome nondisjunction as human females age, aging *C. elegans* exhibits increased chromosome nondisjunction during aging [8], suggesting conserved consequences of germline aging. Several endocrine factors were shown to influence the onset of reproductive senescence in *C. elegans*, including mutations in the *daf-2*/insulin/IGF-1 receptor gene, and the *sma-2/*TGF-β receptor linked Smad transcription factor gene [7], [8], [9]. Inactivating these genes significantly increases reproductive lifespan, and improves the quality of oocytes in later age [7], [8], [9]. Importantly, these genetic factors are also implicated in the regulation of mammalian reproductive lifespan and oocyte quality maintenance [10], [11].

Reproductive aging closely interacts with the decline of somatic health. The positive association between late reproduction and longevity has been observed in various invertebrate and vertebrate animals models and in human population studies. In *Drosophila melanogaster*, long-lived strains can be generated after more than 25 generations of artificial selection of late progeny produced by aged females, which show extended mean lifespan exceeding the maximum lifespan of the original parental strains [12], [13], [14], [15]. In wild chimpanzees, age-associated fertility declines are well correlated with decrease in survival probability [16]. In women, the age at natural menopause is inversely correlated with the rate of all-cause mortality during aging, and females who reproduced their last offspring at advanced age (>50 years) are far more likely to survive to 100 years old [17], [18]. Despite these association studies, the molecular mechanisms underlying the intersection between reproductive aging and somatic aging remain unclear.

Here, we report a comprehensive functional genomic screen to search for genes regulating reproductive senescence in *C. elegans*, and identified 32 gene inactivations that extend reproductive lifespan. Genetic epistasis analysis revealed interactions of some of these genes with insulin/IGF-1 and TGF-β endocrine signaling pathways. We also characterized their requirement and sufficiency in either hermaphrodites or males to promote reproductive longevity. Further demographic analysis showed that 22 of these gene inactivations reduce the mortality rate at young adulthood with or without increasing total life expectancy. Together these new reproductive aging regulators provide an entry point to further characterize the molecular control of reproductive aging, and reveal important mechanistic insights into the intersection between reproductive senescence and organism longevity.

## Results

### Genome-wide RNAi screen for clones extending reproductive lifespan

Wild type *C. elegans* hermaphrodites produce progeny for 7 days at 20°C or for 3 days at 26°C, before they undergo reproductive senescence and cease reproduction. To identify genetic regulators of reproductive aging, we designed a genome-wide RNAi screen to identify those gene inactivations that postpone reproductive senescence in *C. elegans* hermaphrodites. To facilitate high-throughput screening, we used a temperature-sensitive embryonic lethal mutation in a collagen gene, *sqt-3(e2117)*, to destroy all progeny produced during the normal phase of *C. elegans* reproduction so that we could observe only those viable progeny produced after normal reproductive senescence. In the RNAi screen (Figure S1A), *C. elegans* genes were inactivated one gene per well by feeding an *Escherichia coli* strain expressing an approximately one kilobase *C. elegans* segment of double stranded RNA to the *sqt-3(e2117)* mutant animals starting at the first larval stage (L1). The animals were raised at 15°C during larval development, and shifted to the 26°C non-permissive temperature at the fourth larval stage (L4). At this non-permissive temperature, the eggs that are laid during the normal reproductive period are killed by the temperature-sensitive *sqt-3* aberrant collagen mutation. After the normally 3-day reproductive period at 26°C, the adults were shifted back to 15°C on the fourth day, and screened for the presence of live progeny in the well three days later. As expected, we detected no live progeny from the control animals that were fed with bacteria expressing no dsRNA or “non-scoring” dsRNAs (Figure S1B). Reduction of *daf-2* activity by mutation delays the onset of reproductive senescence and extends healthy reproductive lifespan [7]. As a positive control, we scored about 15 live progeny generated per well by the normally reproductively senescent adults fed with *daf-2* RNAi bacteria (Figure S1B). This proves the principle of the genomic screening strategy to systemically explore the regulatory mechanisms of reproductive senescence. By screening 18,413 such dsRNAs, we could survey 94% of *C. elegans* genome for gene inactivations that like *daf-2* gene inactivation delay reproductive senescence.

**Figure 1:**
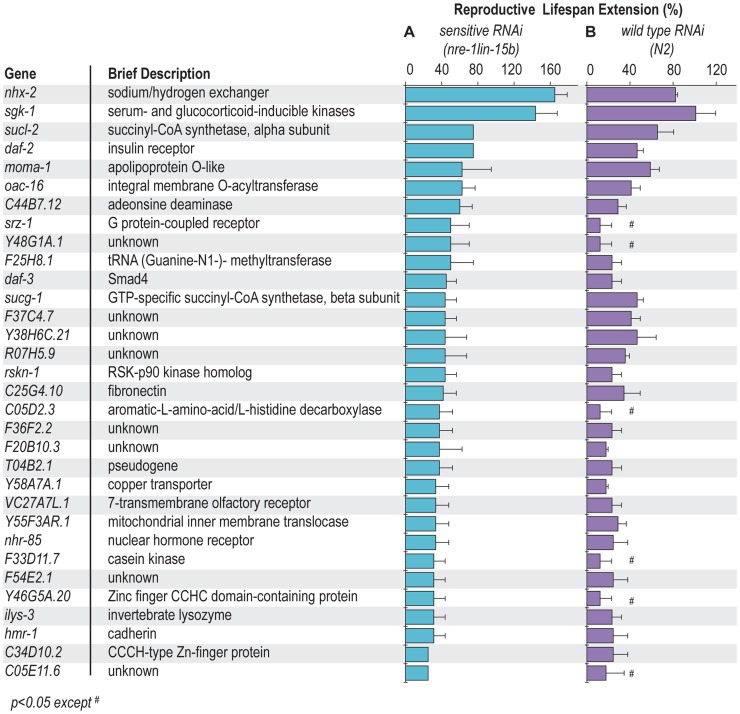
Gene inactivations extending reproductive lifespan. 32 gene inactivations extend reproductive lifespan more than 25% in the RNAi hypersensitive strain, *nre-1(hd20)lin-15b(hd126)* (A). 26 of those gene inactivations also significantly increase reproductive lifespan of wild type (*N2*) (B). The other six gene inactivations promote reproductive longevity only in the *nre-1(hd20)lin-15b(hd126)* strains. They may act in neurons or their RNAi inactivations are only effective in the RNAi hypersensitive background. The average of three independent experiments is shown, *p<0.05* except ^#^.

Out of 18,413 gene inactivations screened, 58 candidates were identified that extend reproductive longevity of *sqt-3* mutant animals (Figure S1C and Table S1). These gene inactivations were further examined in wild type and in an RNAi hypersensitive strain, *nre-1(hd20)lin-15b(hd126)* where RNAi potency is enhanced, especially in neurons [19]. We found that 32 gene inactivations prolong reproductive lifespan by more than 25% in the *nre-1(hd20)lin-15b(hd126)* background (Figure 1A and Table S2), and increase late reproduction of progeny after the first 3 days of adulthood (Figure S2). Twenty-six of the gene inactivations extend reproductive lifespan nearly equivalently in the enhanced neuronal RNAi background and in wild type; the 6 genes that show phenotypes only in the enhanced neuronal RNAi strain background may act in neurons because wild type *C. elegans* generally does not silence neuronal gene functions by RNAi (Figure 1B and Table S3).

One manner by which gene inactivations can appear to increase reproductive longevity is if that gene inactivation slows down the developmental process. That is, if development of the animal or its germline during larval stages is progressing at a decreased rate, the onset of reproduction as well as its cessation may be delayed. On the other hand, if time to initial reproduction is normal, but the cessation of reproduction is delayed, the gene is more likely to regulate reproductive senescence. To distinguish these two possibilities, we examined the time needed to develop from the L1 stage to the onset of reproduction in adulthood for each of the gene inactivations. Nearly all of the reproductive senescence candidate gene inactivations have a normal developmental rate (Figure S3), suggesting that metabolic rate or other gross features of developmental control are not affected by these gene inactivations. Three gene inactivations prolong the developmental time to adulthood, but to a much lesser extent than their effects on the reproductive lifespan extension (Figure S3). Therefore, most of the identified genes indeed affect the program of reproductive senescence, and their effects on reproductive longevity are not simply the result of a developmental delay.

### Genetic classification of genetic regulators of reproductive longevity

**Figure 2:**
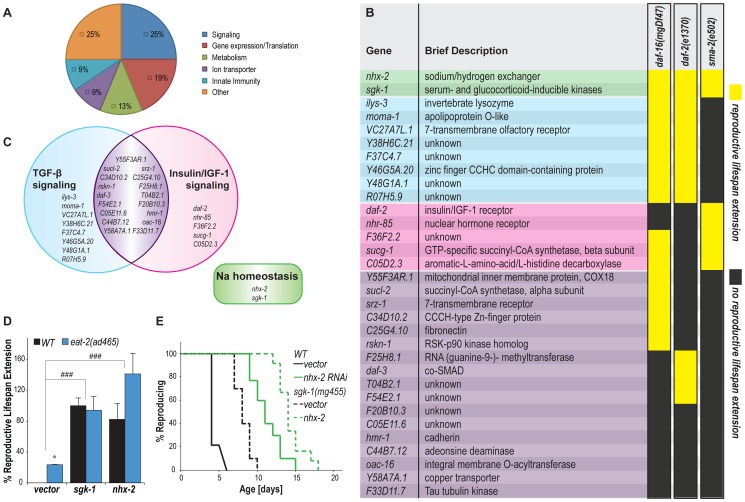
Functional classification of reproductive longevity regulatory genes. (A) Identified reproductive longevity regulatory genes are placed into different groups based on their functional annotation using DAVID bioinformatics analysis. (B) The genetic interaction between the identified genes and insulin/IGF-1 and TGF-β signaling pathways. The identified genes were inactivated in *daf-2(e1370);sqt-3(e2117)*, *daf-16(mgDf47);sqt-3(e2117)* or *sma-2(e502);sqt-3(e2117)* mutants and examined for their effects on the onset of reproductive senescence. Yellow highlights gene inactivations that delay reproductive senescence in the mutants. Dark gray shows gene inactivations that fail to affect reproductive senescence of the mutants. All the experiments were performed at least twice independently. (C) *nhx-2* and *sgk-1*, two regulators of sodium reabsorption, modulate reproductive senescence independently of either insulin/IGF-1 or TGF-β signaling. 17 of the identified genes interact with both pathways. Eight and five genes function specifically in the TGF-β and the insulin/IGF-1 signaling pathway, respectively. (D) *nhx-2* and *sgk-1* regulate reproductive lifespan additively with caloric restriction. *sgk-1* and *nhx-2* were inactivated in the *eat-2(ad465)* mutant, a genetic model of caloric restriction in *C. elegans*. Compared to wild type (*N2*), the *eat-2(ad465)* mutant reproduces 24% longer, * *p<0.05*. RNAi inactivation of either *sgk-1* or *nhx-2* further enhances the reproductive lifespan extension in the *eat-2(ad465)* mutant, ### *p<0.001*. The average of three independent experiments is shown. (E) Inactivation of *nhx-2* and *sgk-1* are additive on reproductive lifespan extension. The *sgk-1(mg455)* null mutation and the *nhx-2* RNAi inactivation prolong reproductive lifespan by 85% and 154%, respectively. The *nhx-2* RNAi inactivation further extends the reproductive lifespan of the *sgk-1* mutant by 75%. *p<0.0001* in all cases.

Functional annotation analysis classified these newly identified genes into different categories, such as signaling transduction, gene expression/translation control, metabolic maintenance, ion transport and innate immunity defense (Figure 2A). To further understand the regulatory mechanisms of these genes, we examined their interactions with the known reproductive longevity pathways regulated by insulin/IGF-1 and TGF-β signaling [7], [8], [9]. Mutations in the insulin/IGF-1 receptor, *daf-2(e1370)*, cause increased reproductive lifespan, which is fully suppressed by a mutation in the negatively regulated FoxO transcription factor, *daf-16(mgDf47)* [7]. To analyze the functions of the identified genes in insulin/IGF-1 signaling, we inactivated the newly identified reproductive senescence genes in the *sqt-3(e2117)* strains carrying either *daf-2(e1370)* or *daf-16(mgDf47)* mutation, performed the similar temperature switching procedure as shown above, and examined whether reproductive senescence could be delayed. As shown in Figure 2B, ten gene inactivations delay reproductive senescence in the background with either *daf-2* or *daf-16* mutation, suggesting that these genes regulate reproductive senescence independently from insulin/IGF-1 signaling. Among the other 22 genes, nine of them act independently of *daf-16*, but their inactivations have no additive effects with the *daf-2* mutation; the effects of four genes are dependent on *daf-16*, but are additive to *daf-2*; and the other nine gene inactivations fail to delay reproductive senescence in either mutant background. These 22 genes are likely acting in insulin/IGF-1 signaling, but at different positions in the pathway.

TGF-β signaling also regulates *C. elegans* reproductive aging; mutations in *sma-2(e502)*, which encodes the TGF-β-receptor-regulated Smad transcription factor, increase reproductive lifespan more than two-fold [8], [9]. We found that five of the identified gene inactivations interacting with insulin/IGF-1 signaling can further extend the reproductive longevity of the *sma-2* mutant, as is also true for the *daf-2* gene inactivation (Figure 2B and 2C, highlighted with pink). On the other hand, eight other gene inactivations delay reproductive senescence of the *daf-2* and *daf-16* mutants, but fail to do so in the *sma-2* mutant (Figure 2B and 2C, highlighted with blue). These data suggest that insulin/IGF-1 and TGF-β signaling modulate the process of reproductive aging in parallel, which is consistent with previous findings [8]. However, our results also show that these two pathways converge on a large group of common downstream targets, considering 17 of the candidate genes interact with both pathways (Figure 2B and 2C, highlighted with purple).

The two most potent effectors, *nhx-2* and *sgk-1*, however act independently of either pathway (Figure 2B and 2C, highlighted with green). In addition to insulin/IGF-1 and TGF-β signaling, caloric restriction using *eat-2* mutants also prolongs reproductive lifespan in *C. elegans* [7]. We found that RNAi inactivation of either *nhx-2* or *sgk-1* further enhances reproductive lifespan extension in the *eat-2(ad465)* mutant (Figure 2D). Because the *eat-2* mutation causes caloric restriction via a disability in the actual ingestion of bacteria, it is unclear if these enhancing gene inactivations act via a regulatory mechanism on the caloric restriction pathway or in parallel to that pathway. An *sgk-1(mg455)* null mutation and *nhx-2* RNAi inactivation exert additive effects on reproductive lifespan extension (Figure 2E), suggesting their parallel functions in regulating reproductive aging. The mammalian homologs of these two genes are the sodium/hydrogen exchanger and the serum-and glucocorticoid-inducible kinase respectively, which both regulate sodium reabsorption [20], [21]. Our results suggest that sodium homeostasis may play a crucial role in the regulation of reproductive senescence.

### Somatic regulation of reproductive longevity

**Figure 3:**
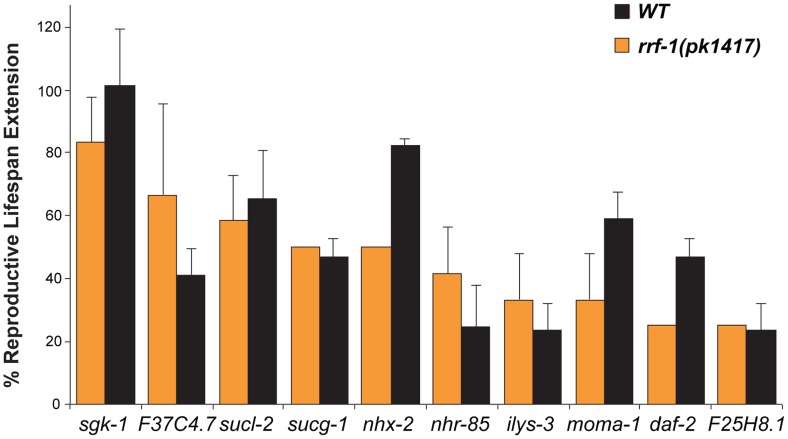
Germline genetic inactivations that prolong reproductive lifespan. In the *rrf-1(pk1417)* mutant, RNAi predominantly operates in the germline. Ten of the identified genes increase reproductive lifespan when inactivated in the *rrf-1* mutants. The extension levels are comparable to that in wild type (*N2)*, except for *daf-2*, *nhx-2* and *moma-1*. The average of three independent experiments is shown, *p<0.05*.

Reproductive activities are under the control of complex interaction between germline and somatic tissues. To test whether the candidate genes function in the germline or in somatic tissues to regulate reproductive longevity, we inactivated the 32 reproductive senescence genes in *rrf-1*(*pk1417*) mutant animals, for which RNAi is predominantly effective only in the germline with a minor effect in the intestine [22], [23]. We found that only 10 gene inactivations prolong the reproductive lifespan of the *rrf-1* mutant animals (Figure 3 and Table sS4). Among them, the effect of *nhx-2/*sodium/hydrogen exchanger, *moma-1/*apolipoprotein O homologue or *daf-2*/insulin-like receptor in the *rrf-1* background is much weaker than that in wild type (Figure 3), suggesting that their functions in somatic tissues are also involved in the regulation of reproductive longevity. Moreover, the other 22 gene inactivations failed to increase reproductive lifespan in the *rrf-1* mutant; those genes likely regulate reproductive longevity systemically via their effects in somatic tissues. Together these studies indicate the significance of somatic functions in the regulation of reproductive senescence.

### Reproductive lifespan extending effects on mated animals

**Figure 4:**
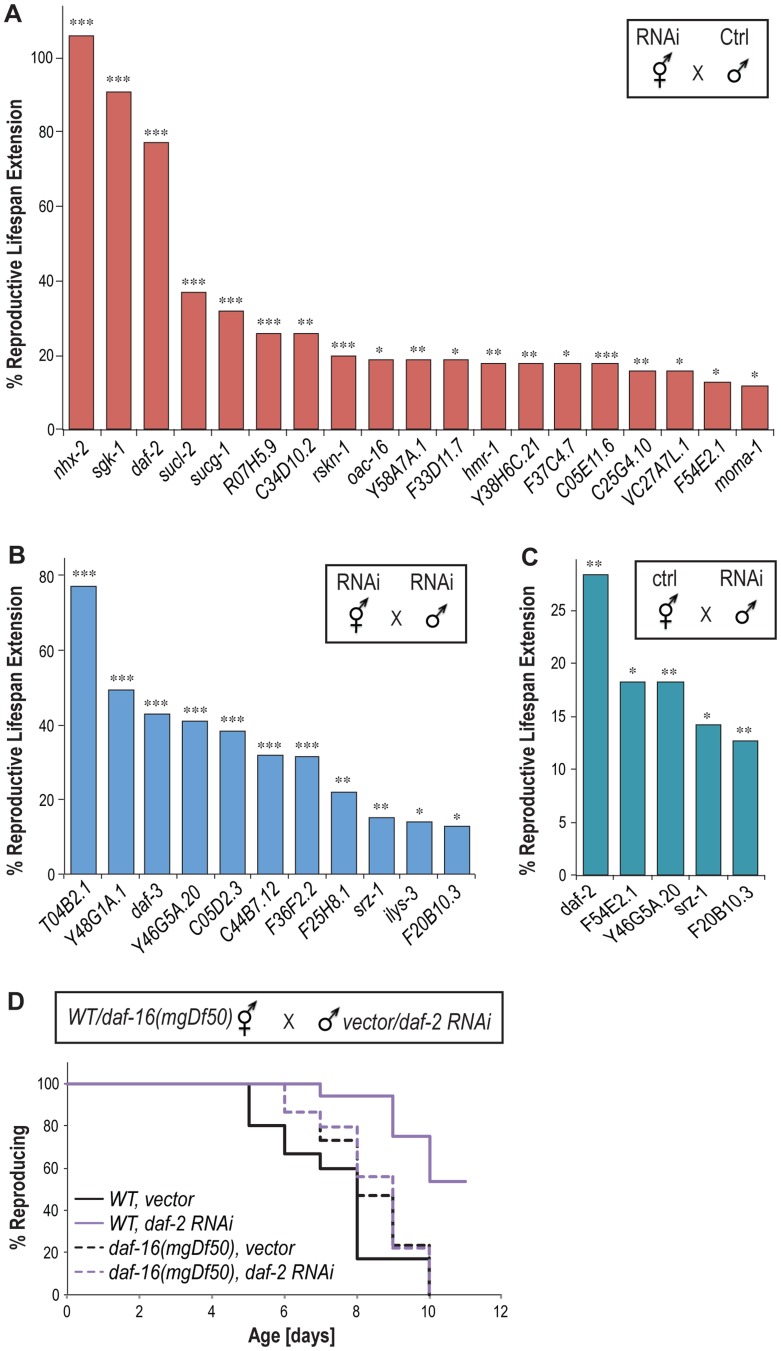
Reproductive longevity extending effects in mated animals. (A) 19 gene inactivations only in hermaphrodites but not males extend the reproductive lifespan of mated hermaphrodites. (B) 11 genes extend reproductive lifespan when inactivated in both hermaphrodites and males. (C) Five gene inactivations in males alone are sufficient to increase the reproductive lifespan of mated hermaphrodites. * p<0.05, ** p<0.01, ***p<0.005. (D) *daf-2* RNAi inactivation only in males prolongs reproductive lifespan of mated wild type hermaphrodites by 37% (*p<0.001*), but fails to do so in the mated *daf-16(mgDf50)* mutant hermaphrodites (*p = 0.79*).

Self-fertilizing *C. elegans* hermaphrodites generate a limited number of sperm by virtue of a pulse of spermatogenesis before a longer run of oogenesis, which constrains their reproductive capacities. However when mated to males which produce far more sperm over a longer period, hermaphrodites use male sperm preferentially, produce double the number of progeny, and reproduce for a much longer time period [7]. To test whether the newly identified genes exert effects on the reproductive longevity of mated animals, we first inactivated these genes in hermaphrodites by RNAi and then crossed those animals with males feeding with control bacteria expressing no dsRNAs. Under this condition, sperm from the males are not affected by RNAi inactivation. We found that 19 genes when inactivated only in mated hermaphrodites are sufficient to promote reproductive longevity (Figure 4A and Table S5), suggesting that these gene inactivations predominantly delay age-associated oocyte senescence.

For the other 13 candidate genes, their inactivations in hermaphrodites alone are not sufficient to enhance reproductive longevity after mating. However, when they are also inactivated in males, 11 of them increase the reproductive lifespan of the mated hermaphrodites (Figure 4B and Table S5), suggesting the requirement of their functions in males to modulate the process of reproductive senescence. To test whether these 11 genes affect sperm production, we examined the effects of their inactivations on brood size in self-fertilizing hermaphrodites where the total number of progeny is determined by sperm quantity. We found that none of these gene inactivations increase total brood size (Figure S4). Therefore, the effect of these genes on reproductive lifespan extension is not simply a result of increased sperm quantity.

We also found five genes whose inactivation only in males are sufficient to prolong the reproductive lifespan of hermaphrodites after mating, including *daf-2*, *F54E2.1*, *Y46G5A.20*, *srz-1* and *F20B10.3* (Figure 4C). Inactivation of *srz-1* and *F20B10.3* in males alone prolongs reproductive lifespan by 14% and 13%, respectively (Figure 4C), which are comparable to the 15% and 13% extension caused by their inactivation in both hermaphrodites and males (Figure 4B). On the other hand, male-only inactivation of *Y46G5A.20* results in 18% reproductive lifespan extension (Figure 4C), which is significantly reduced compared to the 42% extension when *Y46G5A.20* is inactivated in both hermaphrodites and males (Figure 4B). For *daf-2* and *F54E2.1*, their inactivation in either hermaphrodites or males alone is sufficient to prolong reproductive lifespan (Figure 4A and 4C). Interestingly, when mated with the *daf-16(mgDf50)* mutant hermaphrodites, the male-only *daf-2* inactivation failed to prolong reproductive lifespan (Figure 4D). Together, these studies suggest that the effects of the gene inactivations on reproductive longevity occur not only in self-fertilizing hermaphrodites, but also in mated animals. Furthermore, sperm and seminal fluid transferred by mating may induce a humoral response that regulates the process of reproductive senescence in hermaphrodites systemically, and insulin/IGF-1 signaling is involved in this humoral response.

### Effects of reproductive aging regulators on somatic aging-related parameters

**Figure 5:**
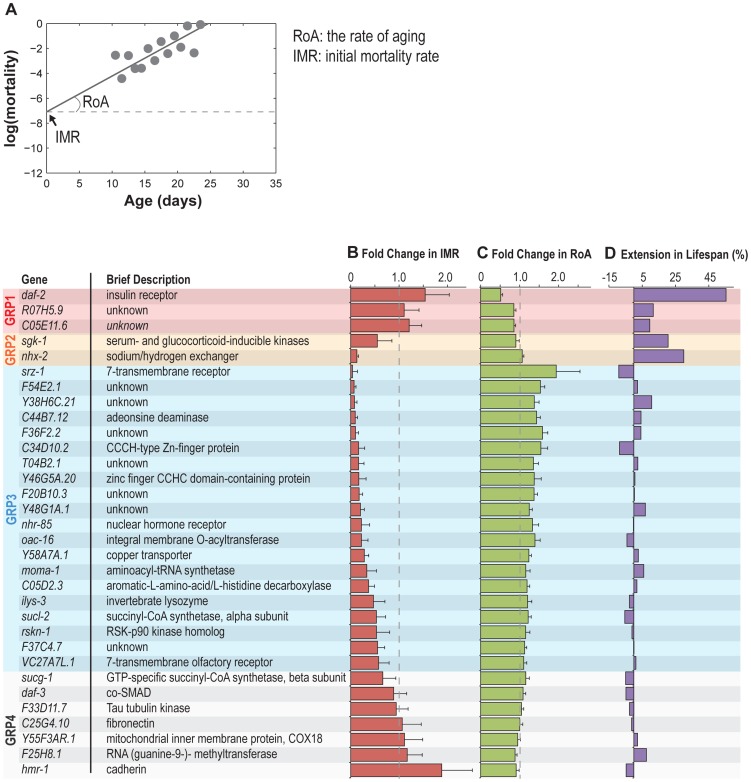
Reproductive longevity regulatory genes modulate somatic longevity. (A) The mortality rate curve of the control animals fed with bacteria expressing no dsRNA. The slope of the curve defines the demographic rate of aging (RoA), and the intercept with the y-axis shows initial mortality rate (IMR). (B–D) The effects of the reproductive senescence genes on somatic aging-related parameters, including IMR (B), RoA (C), and mean lifespan (D). Three gene inactivations including *daf-2*, *R07H5.9* and *C05E11.6* (Group 1), reduce RoA without affecting IMR and increase mean lifespan significantly. Inactivation of *sgk-1* or *nhx-2* (Group 2), two regulators of sodium reabsorption, reduces IMR with no effect on RoA and extends mean lifespan. There are also 20 other gene inactivations (Group 3) that reduce IMR, but increase RoA and do not alter mean lifespan. Six gene inactivations have no effect on somatic longevity (Group 4). For IMR and RoA, the average with standard deviation of three independent experiments is shown; for mean lifespan extension, three independent experiments were combined for analysis.

To characterize the interaction between reproductive aging and somatic aging, we inactivated the 32 genes in the *nre-1(hd20)lin-15b(hd126)* RNAi hypersensitive strain, and examined their effects on organismal lifespan and on age-specific patterns of mortality. In the fitted mortality rate curve under the Gompertz-Makeham model (Figure 5A), the slope of the curve defines the demographic rate of aging (RoA) showing the rate of increase in mortality with age; whereas the intercept with the y-axis defines initial mortality rate (IMR) or “frailty” that represents the mortality rate at the defined time zero, in this case young adulthood and is related to the baseline mortality [24].

**Figure 6:**
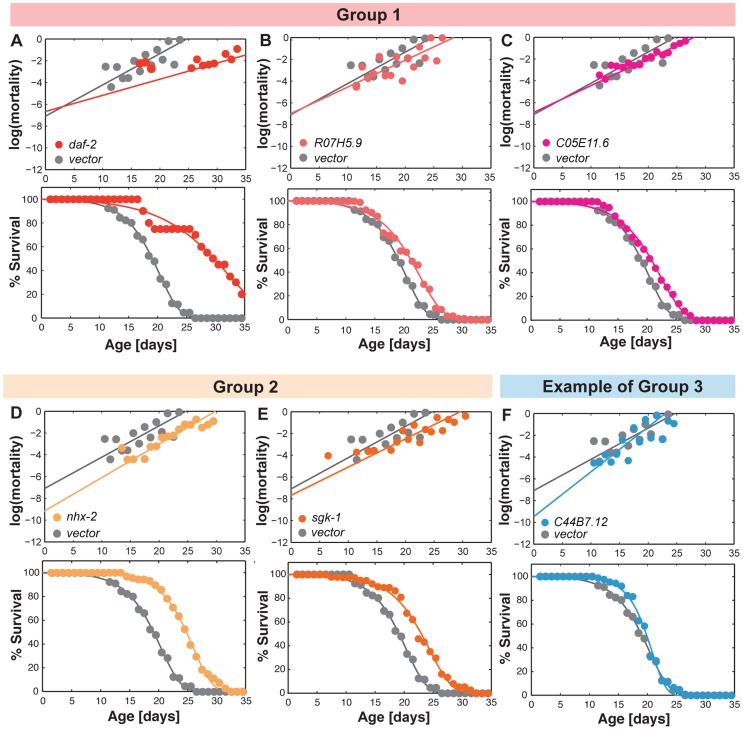
Gene inactivations that promote somatic longevity. (A–C) RNAi inactivation of *daf-2*, *R07H5.9* and *C05E11.6* decrease RoA by 48%, 15% and 14%, respectively, without a significant effect on IMR. The mean lifespan is significantly increased by 55%, 11% and 10%, respectively (*p<0.001*). (D and E) Inactivation of *nhx-2* and *sgk-1* reduce IMR by 87% and 45%, respectively, but does not significantly affect RoA. The mean lifespan is increased by 30% and 21%, respectively (*p<0.001*). (F) *C44B7.12* inactivation reduces IMR by 91%, but increases RoA by 44%. The mean lifespan is not significantly affected. This is one example to represent 20 genes in the Group 3.

We found that the majority (25) of the identified reproductive senescence genes affect somatic mortality trajectories (Figure 5B–D). Based on their effects on different aging-related parameters, these genes can be classified into three groups. First, gene inactivations of three genes, including *daf-2*, *R07H5.9* and *C05E11.6* decrease RoA without affecting IMR (frailty) (Figure 5B–D, Figure 6A–C). As a result, the median and maximum lifespan (defined here as age at 1% estimated survival) are both significantly increased upon their inactivations (Figure 6A–C). Secondly, gene inactivation of the two sodium homeostasis regulatory genes *nhx-2* and *sgk-1*, reduce IMR but not RoA (Figure 5B–D, Figure 6D and 6E). Their inactivations also lead to median and maximum lifespan extension (Figure 6D and 6E), which is consistent with previous findings [25], [26]. The third group includes 20 gene inactivations that decrease IMR, but increase RoA (Figure 5B–D). As a result, these genes have little effect on median lifespan and cause no change in maximum lifespan (Figure 5B–D, one example shown in Figure 6F). Together, these results suggest that most of the gene inactivations that delay reproductive senescence decrease the baseline probability of death and consequently postpone the age-associated rise in mortality.

## Discussion

Our genome scale screen for gene inactivations that delay reproductive senescence identified 32 genes that normally function to mediate reproductive senescence. Characterization of synergies and epistasis of the reproductive lifespan extension induced by these gene inactivations in strains also carrying mutations in insulin/IGF-1 and TGF-β signaling pathways places them into distinct classes. Insulin/IGF-1 and TGF-β signaling are two independent mechanisms to modulate reproductive senescence in *C. elegans*, which are also implicated in regulating mammalian reproductive span and oocyte quality maintenance [10], [11]. Most of our newly identified genes interact with either or both of these two signaling pathways.

*sgk-1* and *nhx-2* are the two most potent effectors identified from the screen and regulate reproductive senescence through a mechanism independent of insulin/IGF-1 signaling, TGF-β signaling or caloric restriction. The mammalian homologues of *sgk-1*, the serum and glucocorticoid activated kinase and *nhx-2*, a sodium/hydrogen exchanger both modulate sodium reabsorption [20], [21]. Thus our data suggest an involvement of sodium homeostasis in the regulation of reproductive senescence in *C. elegans*. Up-regulation of mammalian SGK-1 has been implicated in reproductive failure in women [27]. Therefore, although worms and humans have very distinct reproductive strategies, they may share some common genetic regulatory factors. Twenty-one of the newly identified *C. elegans* genes from the screens are well conserved in human (Table S6), and may provide new insights into the age-associated reproductive senescence in human.

Reproductive success requires proper functions of both oocyte and sperm, and their coordination. In several species including *C. elegans*, sperm can signal to oocyte and to the somatic gonad, and sperm-derived signals are responsible for promoting both oocyte maturation and ovulation [28]. Males can also influence hermaphrodite lifespan in *C. elegans* [29], [30], through secreting diffusible compounds [29]. We found 19 gene inactivations in hermaphrodites only that can promote reproductive longevity. In these cases, mated hermaphrodites use the sperm from the control males not undergoing gene inactivation. Thus the extension of reproductive lifespan under these conditions is caused by the gene inactivations that regulate oocyte activities directly or indirectly. There are 11 genes that require inactivation in both hermaphrodites and males to prolong reproductive lifespan, suggesting the significance of sperm and/or seminal fluid in regulating reproductive aging. Among those 11 genes, only *ilys-3* and *F25H8.1* are sufficient to prolong reproductive lifespan when inactivated in the *rrf-1* mutants, which may exert cell-autonomous effects on the activity of sperm and/or seminal fluid. However, the other nine genes are likely to function in somatic tissues to affect sperm and seminal fluid activity cell-nonautonomously. On the other hand, we also found that five gene inactivations in the male alone, including *daf-2*, *F54E2.1*, *Y46G5A.20*, *srz-1* and *F20B10.3*, are sufficient to prolong the reproductive lifespan of hermaphrodites carrying non-gene inactivated oocyte. These data suggest that sperm produce humoral signals to actively modulate the process of reproductive senescence, and these five genes are likely involved in the sperm humoral signaling. These humoral signals are likely to be secreted steroids and/or peptides; while it is also possible that the sperm or seminal fluid from the males undergoing RNAi treatment may bring gene-inactivating siRNAs into the hermaphrodite. We expect that future characterization of the regulatory mechanisms of those genes will provide insights into the humoral communication between males and females.

Reproductive success is the currency of evolution. To achieve this goal, somatic physiology must be well maintained to support reproductive activities. Thus it is expected that the onset of reproductive senescence would affect somatic maintenance and organism aging. Our data support this, and further reveal that delaying reproductive senescence may have predominant effects on healthy lifespan rather than total lifespan. In our demographic analysis, 3 gene inactivations reduce the mortality rate increase with age (RoA), while 22 gene inactivations decrease the baseline mortality (IMR). IMR measures the initial vulnerability to physiological damage, and its decrease is related to a delay in the onset of chronological aging, or an increase in the initial quality of the organism [24], [31]. Thus, these 22 gene inactivations likely increase healthy life expectancy without necessarily slowing the aging process. Further characterization of these genes may help us find new ways to improve healthy life expectancy, and understand the crucial link between late reproduction and organism longevity during evolution.

## Materials and Methods

### Strains

N2 Bristol was used as the wild-type strain. Other *C. elegans* mutant alleles: *sqt-3(e2117)V, nre-1(hd20)lin-15b(hd126)X, daf-2(e1370)III*, *daf-16(mgDf47)I, sma-2(e502)III*, and *rrf-1(pk1417)I, eat-2(ad465)II, daf-16(mgDf50)I*.

### Genome-wide RNAi reproductive longevity screens

We carried out a large-scale RNAi screen using the *sqt-3(e2117)* strain. RNAi bacteria were cultured 12 h in LB with 50 µg/ml ampicillin and seeded onto 24-well RNAi agar plates containing 5 mM isopropylthiogalactoside (IPTG). The plates were allowed to dry in a laminar flow hood and incubated at room temperature overnight to induce dsRNA expression. Approximately 20∼30 synchronized L1 larvae were placed onto agar plate wells where each *E. coli* strain expressing a distinct *C. elegans* dsRNA corresponding to one gene, and the worms were allowed to develop at 15°C till L4 and shifted to 26°C. After 4 days, the worms were shifted back to 15°C and scored for progeny production after 2∼3 more days of incubation. Worms feeding on bacteria carrying the empty vector (L4440) were used as control. We routinely screened ∼2000–3000 RNAi clones in one experiment. RNAi wells in which live progeny were observed were scored as positives. These “positive” RNAi clones were retested at least three more times using similar high-throughput screening strategy described above. RNAi clones that were scored as positive in all the rescreening tests were deemed “primary positives”. Primary positives were retested three times in RNAi self-fertilizing reproductive lifespan assays (see below) using *the nre-1(hd20)lin-15b(hd126)* or wild type *N2* strain. The RNAi clones that were scored positive three times in the reproductive lifespan assays define the final list of enhanced reproductive longevity gene inactivations.

### Genetic interaction analysis with *daf-2*, *daf-16* and *sma-2*

*daf-2(e1370);sqt-3(e2117)*, *daf-16(mgDf47));sqt-3(e2117)* and *sma-2(e502);sqt-3(e2117)* double mutants were generated. Approximately 20∼30 synchronized L1 larvae were placed onto agar plate wells where each *E. coli* strain expressing a distinct *C. elegans* dsRNA corresponding to one gene, and the worms were allowed to develop at 15°C till L4 and shifted to 26°C. For *daf-2(e1370);sqt-3(e2117)*, the worms were shifted back to 15°C after 9 days and scored for progeny production after 2∼3 more days of incubation. For *daf-16(mgDf47));sqt-3(e2117)* and *sma-2(e502);sqt-3(e2117)*, the worms were kept at 26°C for 4 days and 9 days, respectively. Worms feeding on bacteria carrying the empty vector (L4440) were used as control, in which no progeny was detected after temperature shifting back to 15°C.

### RNAi self-fertilizing reproductive lifespan assay

RNAi bacteria were prepared as described above in 60 mm RNAi agar plates. Approximately 20∼30 synchronized L1 larvae were placed onto the RNAi containing plates and allowed to develop at 20°C until the L4 stage. For each genotype and RNAi treatment, three plates were prepared. At the L4 stage, 10 larvae from each plate were hand- picked and transferred to a fresh RNAi agar plate containing the same RNAi bacteria. The animals then were kept at 20°C and were transferred to fresh RNAi plates every day. After transferring, the old plates were kept at 20°C and scored for the number of progeny two days later. Reproductive lifespan is defined as the first day of reproduction (reproductive lifespan = 1) to when no progeny were scored. The reproductive lifespan of RNAi-treatment groups are compared with that of the control groups (bacteria carrying the empty vector) using a student T-test.

### RNAi mating reproductive lifespan assay

RNAi bacteria were prepared as described above in 35 mm RNAi agar plates. Synchronized L1 larvae (*nre-1(hd20)lin-15b(hd126*) or *daf-16(mgDf50)*) were placed onto the RNAi containing plates and allowed to develop at 20°C. Next, L4 hermaphrodites were mated to young males (*nre-1(hd20)lin-15b(hd126*) at a 1∶2 ratio for 48 hours before being transferred to individual RNAi containing plates. Successful mating was determined by the production of male progeny each day. The individual animal was transferred to fresh plates daily until no progeny scored for at least two days. For each individual, the last day of live progeny production was recorded as the day of reproduction cessation. For each experiment, at least 10 individual hermaphrodites were included. Statistical analyses were performed using SPSS software (http://www-01.ibm.com/software/analytics/spss/). Each RNAi-treatment population is compared with that of the population treated with control RNAi (bacteria carrying the empty vector) using a log rank test.

### RNAi lifespan assay

RNAi bacteria were prepared as described above in 60 mm RNAi agar plates. Approximately 30∼40 synchronized L1 larvae (*nre-1(hd20)lin-15b(hd126*)) were placed onto RNAi-containing agar plates, allowed to develop at 20°C. The animals then were kept at 20°C and transferred to fresh RNAi plates every two days or every seven days during or past the reproductive period respectively. For each RNAi treatment, 3 plates (around 100 worms) were prepared and scored every day by gentle prodding with a platinum wire to test for viability. Lifespan is defined as the first day of adulthood (adult lifespan = 1) to when they were scored as dead. The same experiment was performed three times independently.

### Demographic analysis

We generated lifetables from N_x_, d_x_, and c_x_, the number entering, dying in, or censored in each age interval, respectively. Probability of death (q_x_) was estimated as q_x_ = d_x_/N_x_, and force of mortality as m_x_ = −ln(1−q_x_). The Gompertz-Makeham model for age-specific mortality M(x) = M_0_⋅exp(G⋅x)+M_∞_, with initial mortality M_0_, rate of aging G, age x, and age-independent mortality M_∞_ can be shown to give the survival proportion S(x) = exp[(M_0_/G)(1−exp(G⋅x)−M_∞_⋅x]. We performed non-linear-least-squares fits to S(x) using a Trust-Region Reflective Newton algorithm implemented by the MATLAB *fit()* function (The MathWorks, Natick, MA). Kaplan-Meier estimates of the cumulative distribution (survival) function were computing using the MATLAB (The Mathworks, Natick, MA) function *ecdf()*. We also performed a log-rank test to compare each RNAi gene inactivation to it's associated vector control within the same experiment, using a Bonferroni step-down multiple testing p-value adjustment.

### Progeny production analysis

10 synchronized L4 hermaphrodite larvae (*nre-1(hd20)lin-15b(hd126*)) were transferred to fresh RNAi plates every day and the number of progeny was counted daily until reproduction cessation. The total number of progeny per hermaphrodite was calculated. The experiments were performed three times at 20°C. The RNAi-treatment groups are compared with the control groups (bacteria carrying the empty vector) using a student T-test.

### Developmental time measurement

10 synchronized L1 larvae (*nre-1(hd20)lin-15b(hd126*)) were allowed to develop at 20°C. After becoming L4, the animals were examined every half hour to note the transition to adulthood (oocytes in the germline). If any, the adult individual would be removed from the population. The time from L1 to adulthood when the animal was removed was recorded for each individual. The RNAi-treatment groups are compared with the control groups (bacteria carrying the empty vector) using a student T-test.

## Supporting Information

Figure S1 Design of genome-wide RNAi screen for reproductive longevity. (A) The scheme of RNAi screening using *sqt-3(e2117)* temperature-sensitive mutants. (B) Validation of the screening method by *daf-2* RNAi. After a series of temperature-shift steps as shown in (A), no live progeny were scored from the control worms fed with bacteria expressing no dsRNA. In contrast, approximately 15 live progeny were detected from each well containing about 20 worms fed with *daf-2* RNAi bacterial clones. The average of three independent experiments is shown, *p<0.0001*. (C) The working flow of primary screens and subsequent retests.
(PDF)

Figure S2 The effects of candidate gene inactivations on daily reproductive pattern. The number of progeny was measured in the RNAi inactivated self-fertilizing *nre-1(hd20)lin-15b(hd126)* hermaphrodites. Compared to the vector control, late progeny production is significantly increased by RNAi inactivation of *nhx-2*, *sgk-1*, *sucl-2* and *daf-2*. The average of three independent experiments is shown.
(PDF)

Figure S3 The effects of candidate gene inactivations on developmental timing. Upon RNAi inactivation, the developmental time were measured in the *nre-1(hd20)lin- 15b(hd126)* strains from L1 to adulthood. Except three genes, *nhx-2*, *srz-1* and *daf-3*, the others do not influence developmental time. The average of 10 animals is shown, *** *p<0.005*.
(PDF)

Figure S4 The effects on total brood size in self-fertilizing hermaphrodites. The total number of progeny was measured in the RNAi inactivated self-fertilizing *nre-1(hd20)lin-15b(hd126)* hermaphrodites. None of the gene inactivations leads to an increase in the brood size. Only two genes, *srz-1* and *ilys-3* significantly reduce the total number of progeny when inactivated by RNAi. The average of three independent experiments is shown (n = 10 in each experiment), * *p<0.05*.
(PDF)

Table S1 Candidate genes regulating reproductive aging from genomic RNAi screens.
(PDF)

Table S2 Summary of reproductive lifespan analyses in self-fertilizing *nre-1(hd20);lin-15b(hd126)* strain. Note: self RLS: Average Reproductive LifeSpan in self-fertilizing hermaphrodites from three independent experiments; s.d.: standard deviation; p-value for student's t-test comparing the RNAi treated group to the vector control.
(PDF)

Table S3 Summary of reproductive lifespan analyses in self-fertilizing wild type *(N2)* strain. Note: self RLS: Average Reproductive LifeSpan in self-fertilizing hermaphrodites from three independent experiments; s.d.: standard deviation; p-value for student's t-test comparing the RNAi treated group to the vector control.
(PDF)

Table S4 Summary of reproductive lifespan analyses in self-fertilizing *rrf-1(pk1417)* strain. Note: self RLS: Average Reproductive LifeSpan in self-fertilizing hermaphrodites from three independent experiments; s.d.: standard deviation; p-value for student's t-test comparing the RNAi treated group to the vector control.
(PDF)

Table S5 Summary of reproductive lifespan analyses in mated *nre-1(hd20);lin-15b(hd126)* strains. **Note:** N: Total number of mated hermaphrodites; Mean RLS: Mean Reproductive LifeSpan in mated hermaphrodites; s.d.: standard deviation; p-value for a log-rank test comparing the RNAi treated group to the vector control using Kaplan-Meier survival analysis.
(PDF)

Table S6 The homolog of the candidate genes in human.
(PDF)
