# Supplementary figures and images for: Gene Pathways That Delay Caenorhabditis elegans Reproductive Senescence
Source: PLoS Genet. 2014 Dec 4;10(12):e1004752. doi: 10.1371/journal.pgen.1004752 (PMC4256158; doi:10.1371/journal.pgen.1004752)

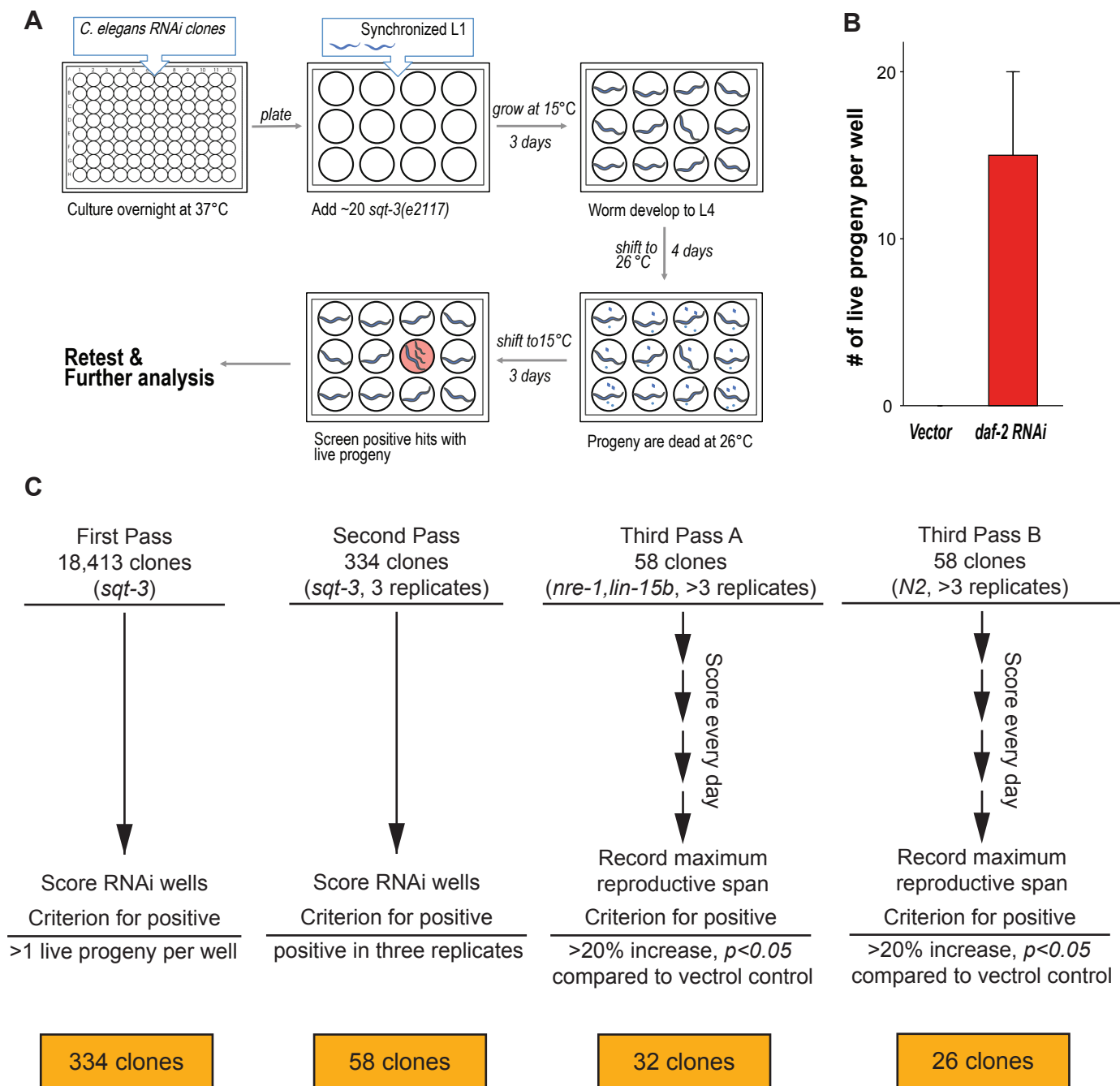

Figure S1 Wang et al

Supplement: Figure S1 — Design of genome-wide RNAi screen for reproductive longevity. (A) The scheme of RNAi screening using sqt-3(e2117) temperature-sensitive mutants. (B) Validation of the screening method by daf-2 RNAi. After a series of temperature-shift steps as shown in (A), no live progeny were scored from the control worms fed with bacteria expressing no dsRNA. In contrast, approximately 15 live progeny were detected from each well containing about 20 worms fed with daf-2 RNAi bacterial clones. The average of three independent experiments is shown, p<0.0001. (C) The working flow of primary screens and subsequent retests. (PDF) [file pgen.1004752.s001.pdf]

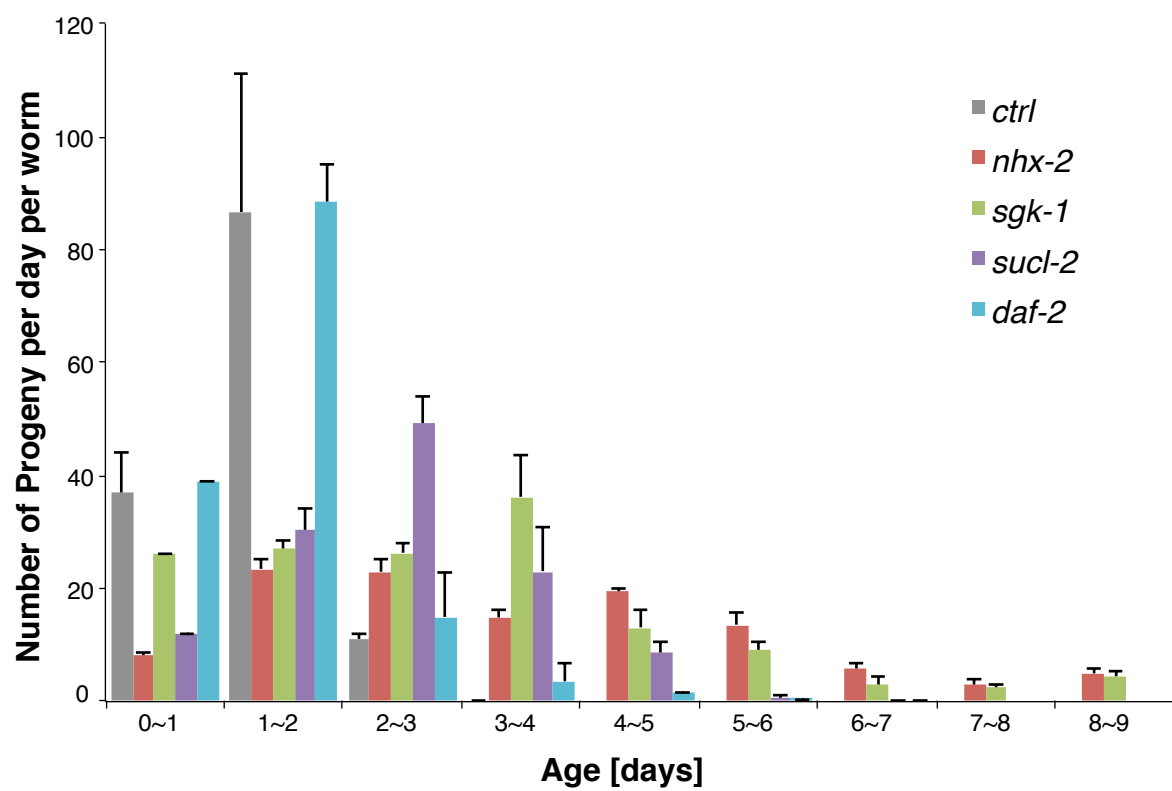

Figure S2 Wang et al

Supplement: Figure S2 — The effects of candidate gene inactivations on daily reproductive pattern. The number of progeny was measured in the RNAi inactivated self-fertilizing nre-1(hd20)lin-15b(hd126) hermaphrodites. Compared to the vector control, late progeny production is significantly increased by RNAi inactivation of nhx-2, sgk-1, sucl-2 and daf-2. The average of three independent experiments is shown. (PDF) [file pgen.1004752.s002.pdf]

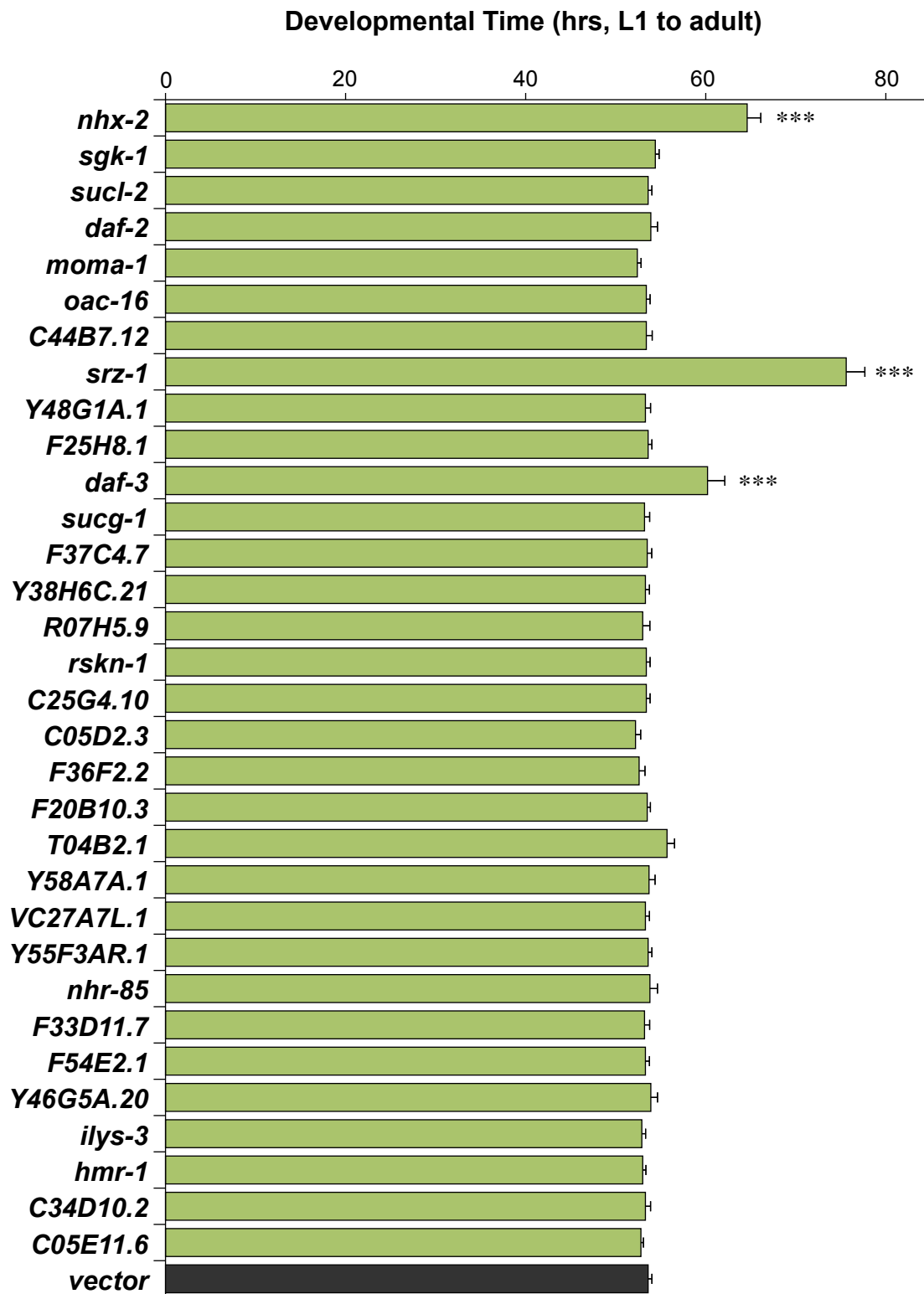

Figure S3 Wang et al

Supplement: Figure S3 — The effects of candidate gene inactivations on developmental timing. Upon RNAi inactivation, the developmental time were measured in the nre-1(hd20)lin- 15b(hd126) strains from L1 to adulthood. Except three genes, nhx-2, srz-1 and daf-3, the others do not influence developmental time. The average of 10 animals is shown, *** p<0.005. (PDF) [file pgen.1004752.s003.pdf]

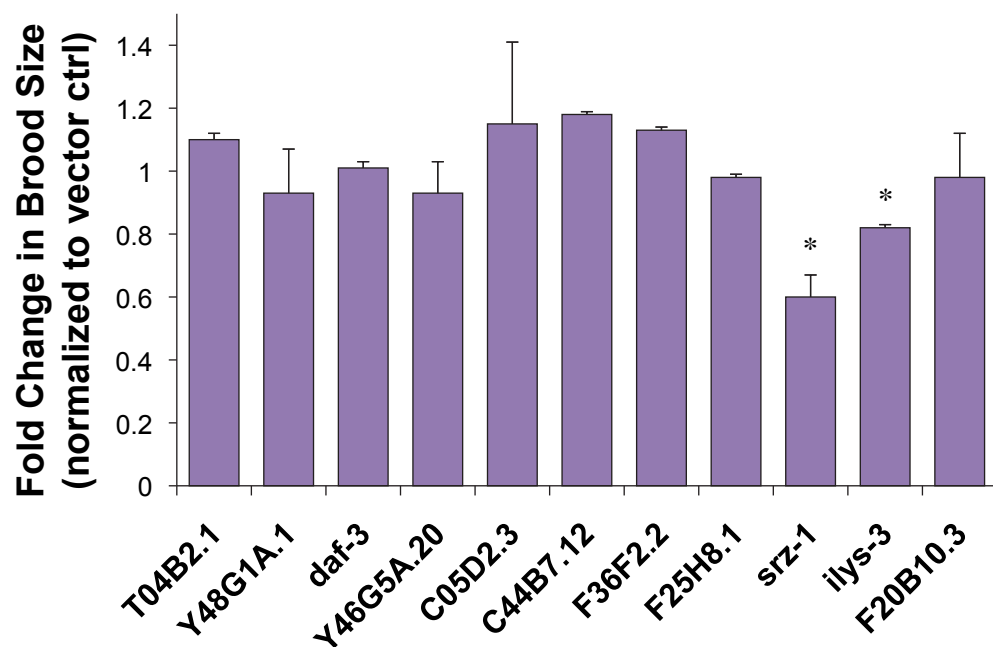

Figure S4 Wang et al

Supplement: Figure S4 — The effects on total brood size in self-fertilizing hermaphrodites. The total number of progeny was measured in the RNAi inactivated self-fertilizing nre-1(hd20)lin-15b(hd126) hermaphrodites. None of the gene inactivations leads to an increase in the brood size. Only two genes, srz-1 and ilys-3 significantly reduce the total number of progeny when inactivated by RNAi. The average of three independent experiments is shown (n = 10 in each experiment), * p<0.05. (PDF) [file pgen.1004752.s004.pdf]
