# Supplementary material for: Gene Pathways That Delay Caenorhabditis elegans Reproductive Senescence
Source: PLoS Genet. 2014 Dec 4;10(12):e1004752. doi: 10.1371/journal.pgen.1004752 (PMC4256158; doi:10.1371/journal.pgen.1004752)
Supplement: Table S1 — Candidate genes regulating reproductive aging from genomic RNAi screens. (PDF) [file pgen.1004752.s005.pdf]

Table S1. Candidate genes regulating reproductive aging from genomic RNAi screens

Gene	Brief Description	Gene	Brief Description
C01A2.1	unknown	*K04G2.7*	unknown
C05D2.3	aromatic-L-amino-acid/L-histidine decarboxylase	*ketn-1*	invertebrate paralog of titin
C05E11.6	unknown	*kin-1*	cAMP-dependent protein kinase, catalytic subunit
C25G4.10	fibronectin	*M28.4*	unknown membrane protein
C34D10.2	CCCH-type Zn-finger protein	*moma-1*	apolipoprotein O-like
C44B7.12	adenosine deaminase	*nhr-85*	nuclear hormone receptor
C45E5.1	sugar phosphatases of the HAD superfamily	*nhr-97*	nuclear hormone receptor
C50F7.4	GTP-specific succinyl-CoA synthetase, beta subunit	*nhx-2*	Na/H exchanger
ceh-8	transcription factor, contains HOX domain	*oac-16*	integral membrane O-acyltransferase
clec-116	c-type lectin	*pif-1*	DNA helicase
clk-1	ubiquinone biosynthesis protein COQ7	*R06F6.7*	unknown
cutl-7	cuticulin precursor	*R07H5.9*	unknown
D1044.5	unknown	*rskn-1*	RSK-p90 kinase homolog
daf-2	insulin/IGF-1 receptor	*sgk-1*	serum- and glucocorticoid-inducible kinase
daf-3	co-SMAD	*srw-130*	7-transmembrane receptor
dcap-2	mRNA decapping enzyme	*srz-1*	7-transmembrane receptor
dgk-5	diacylglycerol kinase	*T04B2.1*	unknown
F20B10.3	unknown	*T05A1.5*	synaptic vesicle transporter
F23H11.3	Succinyl-CoA synthetase, alpha subunit	*T15B12.1*	Ca2+-binding actin-bundling protein fimbrin/plastin
F25H8.1	RNA (guanine-9-)-methyltransferase	*T20B6.1*	receptor-type tyrosine protein phosphatase
F33D11.7	Tau tubulin kinase	*unc-73*	guanine nucleotide exchange factor
F36F2.2	unknown	*VC27A7L.1*	7-transmembrane olfactory receptor
F37C4.7	unknown	*Y38H6C.21*	unknown
F43C11.4	extracellular protein with cysteine rich structures	*Y46G5A.20*	unknown
fbxa-218	F-box protein	*Y48G1A.1*	unknown
grl-8	hedgehog-like protein with the Grl domain	*Y55F3AR.1*	mitochondrial inner membrane protein COX18
hmr-1	cadherins	*Y58A7A.1*	copper transporter
ilys-3	lysozyme protein	*Y61A9LA.4*	Small secreted protein with conserved cysteines
K04G2.2	alpha/beta hydrolase	*ZK909.3*	guanosine polyphosphate pyrophosphohydrolase
