# Supplementary material for: Gene Pathways That Delay Caenorhabditis elegans Reproductive Senescence
Source: PLoS Genet. 2014 Dec 4;10(12):e1004752. doi: 10.1371/journal.pgen.1004752 (PMC4256158; doi:10.1371/journal.pgen.1004752)
Supplement: Table S2 — Summary of reproductive lifespan analyses in self-fertilizing nre-1(hd20);lin-15b(hd126) strain. Note: self RLS: Average Reproductive LifeSpan in self-fertilizing hermaphrodites from three independent experiments; s.d.: standard deviation; p-value for student's t-test comparing the RNAi treated group to the vector control. (PDF) [file pgen.1004752.s006.pdf]

Table S2. Summary of reproductive lifespan analyses in self-fertilizing *nre-1(hd20);lin-15b(hd126)* strain.

Gene	Brief Description	Self RLS	s.d.	p value
ctrl	L4440 vector alone	4.00		
nhx-2	sodium/hydrogen exchanger	10.60	0.55	<0.0001
sgk-1	Serum- and Glucocorticoid-inducible Kinases	9.75	0.96	<0.0001
sucl-2	succinyl-CoA synthetase, alpha subunit	7.00	0.00	<0.0001
daf-2	insulin receptor	7.00	0.00	<0.0001
moma-1	apolipoprotein O-like protein	6.50	1.29	0.0083
oac-16	Integral membrane O-acyltransferase	6.50	0.58	0.0001
C44B7.12	adeonsine deaminase	6.40	0.55	<0.0001
srz-1	G protein-coupled receptor	6.00	0.82	0.0027
Y48G1A.1	unknown	6.00	0.82	0.0027
F25H8.1	tRNA methyltransferase	6.00	1.00	0.0257
daf-3	Smad4	5.80	0.45	<0.0001
sucg-1	GTP-specific succinyl-CoA synthetase, beta subunit	5.75	0.50	0.0004
F37C4.7	unknown	5.75	0.50	0.0004
Y38H6C.21	unknown	5.75	0.96	0.0106
R07H5.9	unknown	5.75	0.96	0.0106
rskn-1	RSK-p90 kinase homolog	5.75	0.50	0.0004
C25G4.10	fibronectin	5.67	0.58	0.0075
C05D2.3	aromatic-L-amino-acid/L-histidine decarboxylase	5.50	0.58	0.0020
F36F2.2	unknown	5.50	0.58	0.0020
F20B10.3	unknown	5.50	1.00	0.0240
T04B2.1	pseudogene	5.50	0.58	0.0020
Y58A7A.1	Copper transporter	5.33	0.58	0.0161
VC27A7L.1	7-transmembrane olfactory receptor	5.33	0.58	0.0161
Y55F3AR.1	mitochondrial inner membrane protein, COX18	5.33	0.58	0.0161
nhr-85	nuclear hormone receptor	5.33	0.58	0.0161
F33D11.7	Casein kinase	5.25	0.50	0.0025
F54E2.1	unknown	5.25	0.50	0.0025
Y46G5A.20	Zinc finger CCHC domain-containing protein	5.25	0.50	0.0025
ilys-3	Invertebrate lysozyme	5.25	0.50	0.0025
hmr-1	cadherin	5.25	0.50	0.0025
C34D10.2	CCCH-type Zn-finger protein	5.00	0.00	<0.0001
C05E11.6	unknown	5.00	0.00	<0.0001

Note: self RLS: Average Reproductive LifeSpan in self-fertilizing hermaphrodites from three independent experiments; s.d.: standard deviation; p-value for student's t-test comparing the RNAi treated group to the vector control
