# Supplementary material for: Gene Pathways That Delay Caenorhabditis elegans Reproductive Senescence
Source: PLoS Genet. 2014 Dec 4;10(12):e1004752. doi: 10.1371/journal.pgen.1004752 (PMC4256158; doi:10.1371/journal.pgen.1004752)
Supplement: Table S3 — Summary of reproductive lifespan analyses in self-fertilizing wild type (N2) strain. Note: self RLS: Average Reproductive LifeSpan in self-fertilizing hermaphrodites from three independent experiments; s.d.: standard deviation; p-value for student's t-test comparing the RNAi treated group to the vector control. (PDF) [file pgen.1004752.s007.pdf]

Table S3. Summary of reproductive lifespan analyses in self-fertilizing wild type *(N2)* strain.

Gene	Brief Description	Self RLS	s.d.	p value
ctrl	L4440 vector alone	5.33	0.58	
nhx-2	sodium/hydrogen exchanger	10.33	1.15	0.0033
sgk-1	Serum- and Glucocorticoid-inducible Kinases	11.33	0.58	0.0003
sucl-2	succinyl-CoA synthetase, alpha subunit	9.33	0.58	0.0015
daf-2	insulin receptor	8.33	1.15	0.0232
moma-1	apolipoprotein O-like protein	9.00	1.00	0.0075
oac-16	Integral membrane O-acyltransferase	8.00	1.00	0.0249
C44B7.12	adeonsine deaminase	7.33	1.15	0.0890
srz-1	G protein-coupled receptor	6.33	0.58	0.2302
Y48G1A.1	unknown	6.33	0.58	0.2302
F25H8.1	tRNA methyltransferase	7.00	1.00	0.0161
daf-3	Smad4	7.00	1.00	0.0161
sucg-1	GTP-specific succinyl-CoA synthetase, beta subunit	8.33	1.15	0.0158
F37C4.7	unknown	8.00	1.00	0.0161
Y38H6C.21	unknown	8.33	1.53	0.0335
R07H5.9	unknown	7.67	0.58	0.0078
rskn-1	RSK-p90 kinase homolog	7.00	1.00	0.0468
C25G4.10	fibronectin	7.67	1.03	0.0386
C05D2.3	aromatic-L-amino-acid/L-histidine decarboxylase	6.33	0.58	0.1012
F36F2.2	unknown	7.00	0.50	0.0368
F20B10.3	unknown	6.67	0.58	0.0474
T04B2.1	pseudogene	7.00	1.00	0.0468
Y58A7A.1	Copper transporter	6.67	0.58	0.0474
VC27A7L.1	7-transmembrane olfactory receptor	7.00	1.00	0.0468
Y55F3AR.1	mitochondrial inner membrane protein, COX18	7.33	1.00	0.0450
nhr-85	nuclear hormone receptor	7.00	0.00	0.0075
F33D11.7	Casein kinase	6.33	0.58	0.1012
F54E2.1	unknown	7.00	0.00	0.0075
Y46G5A.20	Zinc finger CCHC domain-containing protein	6.33	0.58	0.1012
ilys-3	Invertebrate lysozyme	7.00	1.00	0.0468
hmr-1	cadherin	7.00	0.00	0.0075
C34D10.2	CCCH-type Zn-finger protein	7.00	0.00	0.0075
C05E11.6	unknown	6.67	1.15	0.1481

Note: self RLS: Average Reproductive LifeSpan in self-fertilizing hermaphrodites from three independent experiments; s.d.: standard deviation; p-value for student’s t-test comparing the RNAi treated group to the vector control
