# Supplementary material for: Gene Pathways That Delay Caenorhabditis elegans Reproductive Senescence
Source: PLoS Genet. 2014 Dec 4;10(12):e1004752. doi: 10.1371/journal.pgen.1004752 (PMC4256158; doi:10.1371/journal.pgen.1004752)
Supplement: Table S4 — Summary of reproductive lifespan analyses in self-fertilizing rrf-1(pk1417) strain. Note: self RLS: Average Reproductive LifeSpan in self-fertilizing hermaphrodites from three independent experiments; s.d.: standard deviation; p-value for student's t-test comparing the RNAi treated group to the vector control. (PDF) [file pgen.1004752.s008.pdf]

Table S4. Summary of reproductive lifespan analyses in self-fertilizing *rrf-1(pk1417)* strain.

Gene	Brief Description	Self RLS	s.d.	p value
ctrl	L4440 vector alone	4.00	0.00	
nhx-2	sodium/hydrogen exchanger	10.33	1.15	0.0033
sgk-1	Serum- and Glucocorticoid-inducible Kinases	7.33	0.58	0.0006
sucl-2	succinyl-CoA synthetase, alpha subunit	6.33	0.58	0.0022
daf-2	insulin receptor	5.00	0.00	<0.0001
moma-1	apolipoprotein O-like protein	5.00	0.00	<0.0001
F25H8.1	tRNA methyltransferase	5.00	0.00	<0.0001
sucg-1	GTP-specific succinyl-CoA synthetase, beta subunit	6.00	0.00	<0.0001
F37C4.7	unknown	6.67	1.15	0.0161
nhr-85	nuclear hormone receptor	5.33	0.58	0.0161
ilys-3	Invertebrate lysozyme	5.33	0.58	0.0161

Note: self RLS: Average Reproductive LifeSpan in self-fertilizing hermaphrodites from three independent experiments; s.d.: standard deviation; p-value for student's t-test comparing the RNAi treated group to the vector control
