# Supplementary material for: Gene Pathways That Delay Caenorhabditis elegans Reproductive Senescence
Source: PLoS Genet. 2014 Dec 4;10(12):e1004752. doi: 10.1371/journal.pgen.1004752 (PMC4256158; doi:10.1371/journal.pgen.1004752)
Supplement: Table S5 — Summary of reproductive lifespan analyses in mated nre-1(hd20);lin-15b(hd126) strains. Note: N: Total number of mated hermaphrodites; Mean RLS: Mean Reproductive LifeSpan in mated hermaphrodites; s.d.: standard deviation; p-value for a log-rank test comparing the RNAi treated group to the vector control using Kaplan-Meier survival analysis. (PDF) [file pgen.1004752.s009.pdf]

Table S5. Summary of reproductive lifespan analyses in mated *nre-1(hd20);lin-15b(hd126)* strains.

Gene	Brief Description	N	Mean RLS	s.d.	p value
ctrl	L4440 vector	36	6.80	0.25	
nhx-2	sodium/hydrogen exchanger	12	14.00	0.25	<0.001
sgk-1	Serum- and Glucocorticoid-inducible Kinases	12	13.00	0.15	<0.001
daf-2	insulin receptor	12	12.00	0.14	<0.001
sucl-2	succinyl-CoA synthetase, alpha subunit	12	9.30	0.13	<0.001
sucg-1	GTP-specific succinyl-CoA synthetase, beta subunit	12	9.00	0.20	<0.001
R07H5.9	unknown	12	8.60	0.27	<0.001
C34D10.2	CCCH-type Zn-finger protein	12	8.60	0.48	0.005
rskn-1	RSK-p90 kinase homolog	12	8.13	0.23	0.004
oac-16	Integral membrane O-acyltransferase	12	8.10	0.41	0.012
Y58A7A.1	Copper transporter	12	8.10	0.24	0.005
F33D11.7	Casein kinase	12	8.10	0.51	0.042
F37C4.7	unknown	12	8.00	0.15	0.012
Y38H6C.21	unknown	12	8.00	0.27	0.009
hmr-1	cadherin	12	8.00	0.33	0.006
C05E11.6	unknown	12	8.00	0.11	<0.001
C25G4.10	fibronectin	12	7.90	0.35	0.009
VC27A7L.1	7-transmembrane olfactory receptor	12	7.89	0.48	0.019
F54E2.1	unknown	12	7.67	0.42	0.021
moma-1	apolipoprotein O-like protein	12	7.60	0.37	0.039
ctrl	L4440 vector	25	6.31	0.38	
T04B2.1	pseudogene	10	11.16	0.75	0.001
Y48G1A.1	unknown	15	9.43	0.75	0.001
daf-3	Smad4	10	9.01	0.87	0.004
Y46G5A.20	Zinc finger CCHC domain-containing protein	12	8.90	0.48	0.001
C05D2.3	aromatic-L-amino-acid/L-histidine decarboxylase	10	8.74	0.47	0.001
C44B7.12	adeonsine deaminase	11	8.35	0.39	<0.001
F36F2.2	unknown	12	8.31	0.50	0.004
F25H8.1	tRNA methyltransferase	12	7.70	0.45	0.006
srz-1	G protein-coupled receptor	12	7.24	0.13	0.005
ilys-3	Invertebrate lysozyme	12	7.19	0.16	0.014
F20B10.3	unknown	12	7.15	0.26	0.030
ctrl	L4440 vector	37	5.41	0.34	
daf-2	insulin receptor	10	7.00	0.44	0.009
F54E2.1	unknown	10	6.40	0.44	0.036
Y46G5A.20	Zinc finger CCHC domain-containing protein	22	6.40	0.30	0.007
srz-1	G protein-coupled receptor	23	6.18	0.25	0.022
F20B10.3	unknown	23	6.09	0.30	0.006

Note: 1. N: Total number of mated hermaphrodites; Mean RLS: Mean Reproductive LifeSpan in mated hermaphrodites; s.d.: standard deviation; p-value for a log-rank test comparing the RNAi treated group to the vector control using Kaplan-Meier survival analysis.
2. RNAi inactivation only in hermaphrodites
3. RNAi inactivation in both hermaphrodites and males
4. RNAi inactivation only in males
