# Supplementary material for: Gene Pathways That Delay Caenorhabditis elegans Reproductive Senescence
Source: PLoS Genet. 2014 Dec 4;10(12):e1004752. doi: 10.1371/journal.pgen.1004752 (PMC4256158; doi:10.1371/journal.pgen.1004752)
Supplement: Table S6 — The homolog of the candidate genes in human. (PDF) [file pgen.1004752.s010.pdf]

Table S6 The homolog of the candiate genes in human

Gene	Brief Description	Human Homolog
nhx-2	Na/H exchanger	yes
sgk-1	serum- and glucocorticoid-inducible kinases	yes
F23H11.3	succinyl-CoA synthetase, alpha subunit	yes
daf-2	insulin receptor	yes
moma-1	apolipoprotein O-like	yes
oac-16	integral membrane O-acyltransferase	no
C44B7.12	adeonsine deaminase	yes
srz-1	7-transmembrane receptor	yes
Y48G1A.1	unknown	no
F25H8.1	RNA (guanine-9-)- methyltransferase	yes
daf-3	co-SMAD	yes
C50F7.4	GTP-specific succinyl-CoA synthetase, beta subunit	yes
F37C4.7	unknown	no
Y38H6C.21	unknown	no
R07H5.9	unknown	no
rskn-1	RSK-p90 kinase homolog	yes
C25G4.10	fibronectin	yes
C05D2.3	aromatic-L-amino-acid/L-histidine decarboxylase	yes
F36F2.2	unknown	no
F20B10.3	unknown	no
T04B2.1	unknown	no
Y58A7A.1	copper transporter	yes
VC27A7L.1	7-transmembrane olfactory receptor	yes
Y55F3AR.1	mitochondrial inner membrane protein COX18	yes
nhr-85	nuclear hormone receptor	yes
F33D11.7	Tau tubulin kinase	yes
F54E2.1	unknown	no
Y46G5A.20	zinc finger CCHC domain-containing protein	yes
ilys-3	invertebrate lysozyme	no
hmr-1	cadherin	yes
C34D10.2	CCCH-type Zn-finger protein	yes
C05E11.6	unknown	no
